# Downregulation of MCF2L Promoted the Ferroptosis of Hepatocellular Carcinoma Cells through PI3K/mTOR Pathway in a RhoA/Rac1 Dependent Manner

**DOI:** 10.1155/2022/6138941

**Published:** 2022-10-25

**Authors:** Si-cong Huang, Yi-mei Chen, Ying-yu Hu, Yong-jie Shi, Qi-wen Xiao, Zhe Li, Jia-le Kang, Qiang Zhou, Gang Shen, Hong-yun Jia

**Affiliations:** ^1^Department of Clinical Laboratory, The Second Affiliated Hospital of Guangzhou Medical University, Guangzhou, Guangdong, China; ^2^Department of Healthy Examination, The Second Affiliated Hospital of Guangzhou Medical University, Guangzhou, Guangdong, China; ^3^Department of Business Developement, Zhujiang Hospital of Southern Medical University, Guangzhou, Guangdong, China; ^4^Department of Neurology, The Second Affiliated Hospital of Guangzhou University of Chinese Medicine, Guangzhou, Guangdong, China; ^5^Department of Interventional Radiology and Vascular Anomalies, Children's Hospital, Capital Institute of Pediatrics, Beijing, China

## Abstract

**Methods and Results:**

The levels of MCF2L were detected by PCR and western blotting assay. The effect of MCF2L on ferroptosis was confirmed by MTT, colony formation assay, Brdu, in vivo animal experiment, and the content of Iron, GSH, ROS, and MDA. The underlying mechanisms were explored by PCR, western blotting, and affinity precipitation assay. Our findings demonstrated that MCF2L is remarkedly upregulated in HCC tissues, and sorafenib can induce the levels of MCF2L, suggesting that MCF2L might function in sorafenib resistance of HCC. Further analysis showed that downregulation of MCF2L enhances HCC cell death induced by sorafenib, and ferroptosis inhibitor can reverse this process. Subsequent experiments showed that downregulation of MCF2L elevates the content of Iron, ROS, and MDA, which are all indicators of ferroptosis. Finally, mechanism analysis showed that MCF2L regulates the PI3K/AKT pathway in a RhoA/Rac1 dependent manner.

**Conclusions:**

Our study showed that targeting MCF2L may be a hopeful method to overcome sorafenib-resistance through inducing ferroptosis in HCC.

## 1. Introduction

Hepatocellular carcinoma (HCC) is one of the most common and deadly malignancy worldwide [1]. Most patients are diagnosed at advanced stage, which induces patients suffering chemotherapy [2]. But, multiple patients received chemotherapy results in primary or secondary chemoresistance. The underlying mechanisms of chemoresistance are rather vague, therefore, it is essential to clarify the mechanisms of chemoresistance and to implement a corresponding therapy strategy.

Chemotherapeutic drugs usually induce cell death to therapy cancer [3]. Originally, cell death is divided into three forms according to their characteristics: apoptosis, autophagy, and necrosis [4]. Especially, caspase-dependent apoptosis was regarded as the only form of regulated cell death (RCD), and many anticancer drugs were developed based on apoptosis [5]. However, the efficacy of these drugs has not been satisfactory. Taken sorafenib for example, it is the first targeted drug approved by the FDA for advanced HCC, but it is correlated with seriously adverse side effects and drug resistance [6]. In recent years, scientists have discovered some new ways of cell death, which challenge the traditional understanding of cell death. These newly discovered RCDs have different mechanisms from apoptosis, which can bypass the limitations of apoptosis and provide new opportunities to therapy cancer [7].

Thereinto, ferroptosis has been defined as a novel RCD class of iron-dependent lipid reactive oxygen species (ROS) and lipid peroxidation products [8]. Although ferroptosis takes an indispensable role in cell survival, it has been increasingly considered that several cancer signalings are associated with ferroptosis, which results in cancer cells highly susceptible to ferroptosis [9]. The amount of lipid ROS can be decided through impaired detoxification by glutathione peroxidase 4 (GPX4) [10], or through production of superoxide and hydrogen peroxide by upregulation of nicotinamide adenine dinucleotide phosphate (NADPH) oxidase (NOXs) [8]. Downregulation of GPX4 can induce ferroptosis [11]. However, much remains unclear regarding the genetic determinants of ferroptosis in HCC.

MCF2L belongs to the Dbl family, which is guanine nucleotide exchange factors (GEFs) to work through Rac1 and RhoA, and several reports suggest that specific members of the Dbl family might be mutated in human tumors [12]. MCF2L can regulate cell motility of tumor-derived human breast epithelial cell via activating Cdc42 and Rac1 [13]. Whereas, the expression profile and function of MCF2L in HCC remain to be investigated.

The purpose of the present study is to explore the expression levels and function of MCF2L in HCC.

## 2. Materials and Methods

### 2.1. Cell Culture

HCC cells were purchased from Cell Resource Center, Institute of Basic Medicine, Chinese Academy of Medical Sciences and were cultured using Dulbecco's modified Eagle medium (DMEM, Sigma-Aldrich) containing 10% fetal bovine serum (FBS, HyClone), 100 units/mL penicillin, and 100 mg/mL streptomycin. All cells were kept in a humidified atmosphere containing 5% CO_2_ at 37°C.

### 2.2. Quantitative Real-Time PCR (qRT-PCR) Assay

The total RNA was collected with TRIzol reagent (Invitrogen, USA). And cRNA were subsequently obtained by Transcriptor First Strand cDNA Synthesis Kit (Roche, Germany) following the manufacturer's directions. qPCR was carried out on a 7500 Fast Real time PCR system (Applied Biosystems, USA) with the SYBR Green PCR Kit (Invitrogen, USA). The primers in the study were as follows: MCF2L: forward, 5′- AAACCGAGGCTGCCTTCGATGA-3′; reverse, 5′- TGCCGATGTCTGTGAAGGTTGC-3′. GPX4, forward, 5′- ACAAGAACGGCTGCGTGGTGAA-3′; reverse, 5′- GCCACACACTTGTGGAGCTAGA-3′. NOX1, forward, 5′- GGTTTTACCGCTCCCAGCAGAA-3′; reverse, 5′- CTTCCATGCTGAAGCCACGCTT-3′. NOX2(CYBB), forward, 5′- CTCTGAACTTGGAGACAGGCAAA -3′; reverse, 5′- CACAGCGTGATGACAACTCCAG -3′. NOX3, forward, 5′- CCTGGAAACACGGATGAGTGAG -3′; reverse, 5′- CCTCCCATAGAAGGTCTTCTGC -3′. NOX4, forward, 5′- GCCAGAGTATCACTACCTCCAC -3′; reverse, 5′- CTCGGAGGTAAGCCAAGAGTGT -3′. NOX5, forward, 5′- CCACCATTGCTCGCTATGAGTG -3′; reverse, 5′- GCCTTGAAGGACTCATACAGCC -3′. DUOX1, forward, 5′- TCTCTGGCTGACAAGGATGGCA -3′; reverse, 5′- AGGCGAGACTTTTCCTCAGGAG -3′. DUOX2, forward, 5′- CAATGGCTACCTGTCCTTCCGA -3′; reverse, 5′- GTCCTTGGAGAGGAAGCCATTC -3′. GAPDH, forward, 5′- GTCTCCTCTGACTTCAACAGCG -3′; reverse, 5′- ACCACCCTGTTGCTGTAGCCAA -3′.

### 2.3. The Establishment of Stable Cell Line

The MCF2L shRNA and corresponding scramble shRNA vector were obtained from GeneCopoeia Biotech (Guangzhou, China). For viral packaging, psPAX2 (virus-packaging plasmid), pMD2G (envelope plasmid), and pLKO.1 plasmids were transfected into 293 T cells to produce pseudotyped lentiviral particles. After 48 hours, the particles were collected and used to infect MHCC97H or Huh7 cells overnight. We then used the normal culture media to replace transfection media. We screened the stable cells with DDR2 downregulation using 5 *μ*g/mL puromycin for 7 days.

### 2.4. Western Blotting Assay

Extracted proteins were suffered to western blotting following previously described methods [14]. 10.5% polyacrylamide gel was used to separate protein. And then, the protein was transferred onto PVDF membranes. Subsequently, the protein was probed using primary antibodies and then incubated with horseradish peroxidase (HRP)-conjugated secondary antibody. We used GAPDH as the loading control.

### 2.5. RhoA/Rac1 Activation Assay

The levels of endogenous GTP-bound RhoA and Rac1 were examined by affinity precipitation assay following the previous method [13].

### 2.6. Cell Viability Assay

The cell viability was detected by MTT assay. In brief, 5 × 10^3^ cells were implanted into 96-well culture plated and cultured in a humidified incubator for 12 h. Subsequently, different reagents (50 nM Sorafenib, 2 *μ*M Ferrostatin-1, 3 *μ*M Z-Vad-FMK, 10 *μ*M necrosulfonamide, 10 mM 3-MA) were supplemented into the well and continued culturing for 48 h. 10 mg/mL MTT solution was added into the well for 4 h. And then, 100 *μ*L DMSO was added into the well. Ultimately, the absorbance was examined at 570 nm wave length by the multifunctional enzyme marker (Thermo Fisher Scientific; USA).

### 2.7. Colony Formation Assay

Cells were implanted into 12-well plates and cultured in incubator at 37°C for 12 h. 50 nM sorafenib was added into the well. Ten days later, the cells were fixed using 4% paraformaldehyde and then stained with 0.1% crystal violet. The stained colonies were counted under a light microscope.

### 2.8. Bromodeoxyuridine (Brdu) Labeling and Immunofluorescence

Cells were implanted on coverslips, which were put into 24-well plates. 12 hours later, 50 nM sorafenib was supplemented into the well. The cells were further cultured for 24 hours, subsequently incubated using Brdu for 1 hour, and then stained using anti-Brdu antibody (Upstate Biotechnology, USA). Finally, Brdu images were captured using a laser scamming microscope (Carl Zeiss, Germany).

### 2.9. In Vivo Tumorignesis Assay

Four-week-old BALB/c mice were purchased from GuangDong GemPharmatech Co.,Ltd.(GuangDong, China) and were housed in a specific pathogen-free animal house. Then, 3 × 10^5^ HCC cells were suspended and subcutaneously injected into the flanks of BALB/c nude mice. Ten days later, tumors were considered fully formed. The mice were treated by sorafenib (30 mg/kg/day) by oral gavage. The mice were sacrificed. All procedures were approved by the Institutional Animal Care and Use Committee of The Second Affiliated Hospital of Guangzhou Medical University.

### 2.10. Iron/ROS/MDA/GSH Assay

The relative Iron, ROS, MDA and GSH contents in cell lysates were detected using an Iron Assay Kit (#ab83366, abcam, USA), Total Reactive Oxygen Species (ROS) Assay Kit (#88-5930-74; Thermo Fisher Scientific, USA), Lipid Peroxidation (MDA) Assay Kit (#ab118970; Abcam, USA), and Glutathione (GSH) Assay Kit (#CS0260, Sigma, USA) according to the manufacturer's instructions, respectively.

### 2.11. Statistical Analysis

Statistical analyses were performed using the SPSS version 19.0 statistical software package. The data are present as the mean ± standard deviation. Student's paired *t*-test was used to analyze the statistical difference between paired tissues, and comparisons among more than two groups were analyzed using variance (ANOVA) followed by Dunnett's test. *P* < 0.05 was considered statistically significant.

## 3. Results

### 3.1. MCF2L Is Correlated with Sorafenib-Resistance in HCC

**Figure 1:**
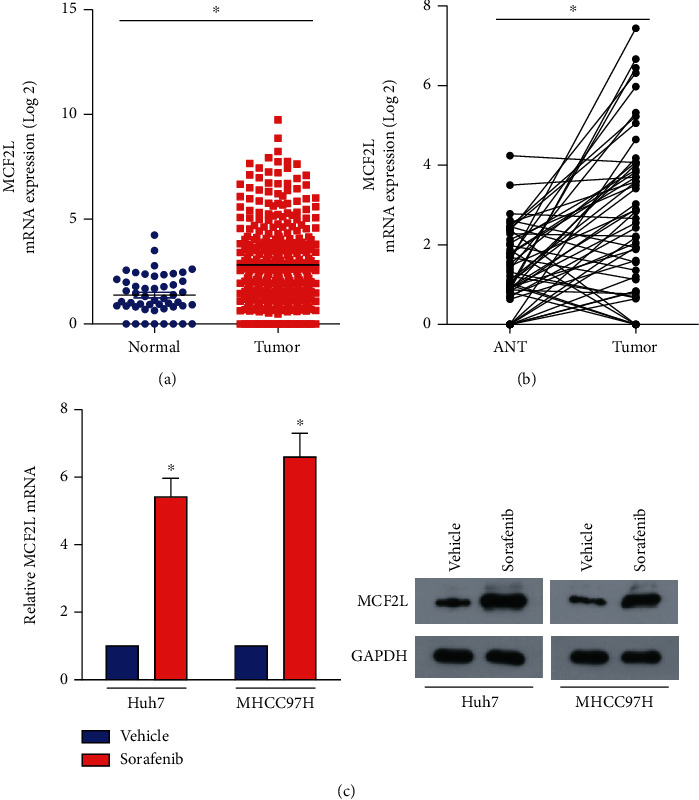
MCFL2 is involved in sorafenib-resistance in HCC. (a) MCF2L is significantly upregulated in HCC through analyzing the data from the public dataset The Cancer Genome Atlas (TCGA; https://cancergenome.nih.gov/). (b) The expression of MCF2L in paired HCC tissues. (c) MCF2L is significantly increased in HCC cells-treated with sorafenib both on mRNA (left panel) and protein (right panel) levels. ANT: adjacent normal tissue; ^∗^*P* < 0.05.

Through analyzing the data from the public dataset The Cancer Genome Atlas (TCGA; https://cancergenome.nih.gov/), we found that MCF2L is significantly upregulated in HCC (Figure 1(a)). Meanwhile, we analyzed its expression profile in paired HCC tissue with data from TCGA. As demonstrated in Figure 1(b), the level of MCF2L is substantial upregulated in HCC tissues relative to the corresponding adjacent normal tissue (ANT). The above analysis suggested that MCF2L takes an indispensable role in HCC progression.

Since sorafenib is the first targeted drug approved by the FDA for advanced HCC, and usually induces drug resistance to result in unsatisfactory efficacy, we investigated whether MCF2L works in sorafenib resistance. As shown in Figure 1(c), MCF2L is remarkedly increases in HCC cells-treated with sorafenib both on mRNA and protein levels, which inferred that MCF2L functioned in sorafenib resistance of HCC.

### 3.2. Downregulation of MCF2L Enhances HCC Cell Death Induced by Sorafenib

**Figure 2:**
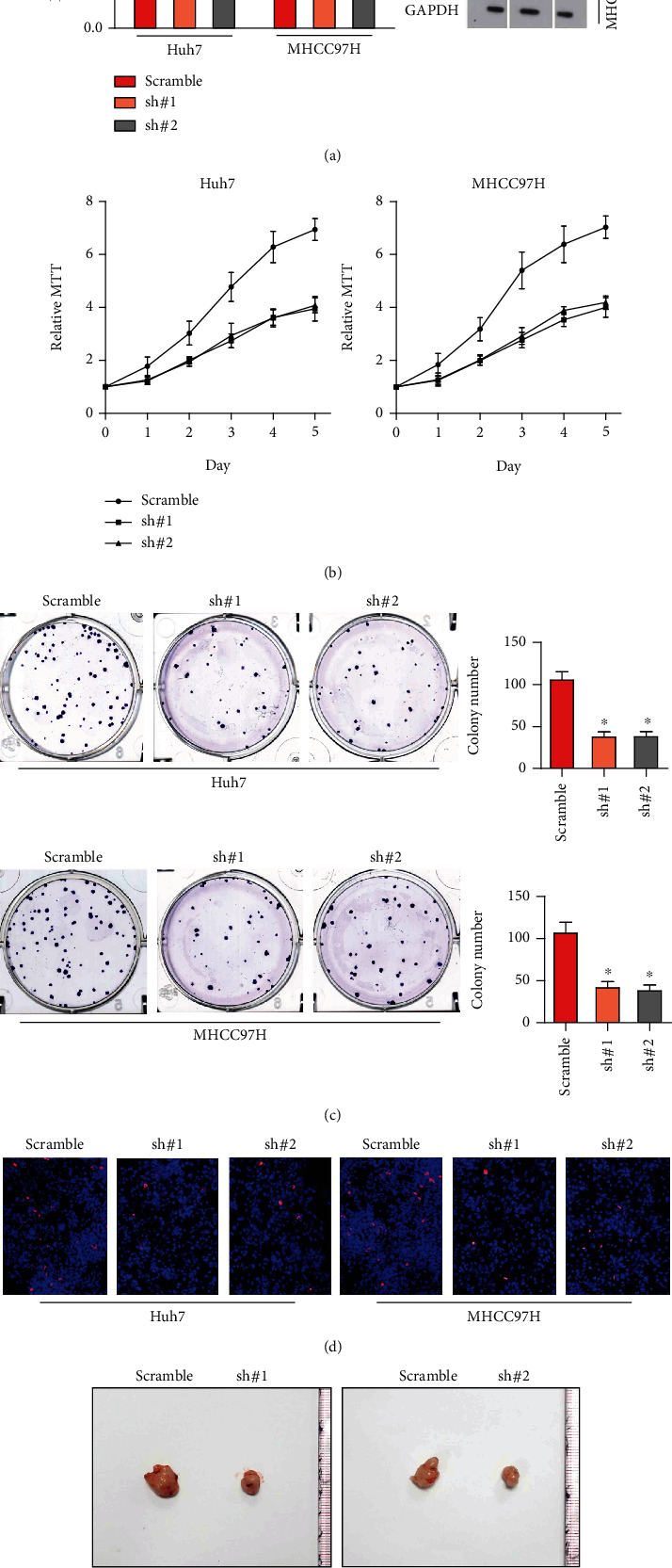
Downregulation of MCF2L enhances HCC cell death induced by sorafenib. (a) The mRNA (left panel) and protein (right panel) levels of MCF2L in indicated stable cell line. (b) MTT assay showed that downregulation of MCF2L enhances HCC cell death induced by sorafenib. (c) The representative image (left panel) and quantitative assay (right panel) of colony formation showed that downregulation of MCF2L enhances HCC cell death induced by sorafenib. (d) The representative images (left panel) and quantitative assay (right panel) of Brdu assay. (e) The in vivo tumorigenesis assay showed downregulation of MCF2L inhibits the tumorigenesis under the treatment of sorafenib ^∗^*P* < 0.05.

In order to further clarify the roles of MCF2L in sorafenib resistance, MCF2L was stably knockdown in Huh7 and MHCC97H using specific shRNA. PCR and western blotting assay illustrated that MCF2L is remarkedly decreased in MCF2L-knockdown cells (Figure 2(a)). MTT (Figure 2(b)) and colony formation (Figure 2(c)) assay showed that downregulation of MCF2L enhances HCC cell death induced by sorafenib. More, Brdu assay was performed. As shown in Figure 2(e), downregulation of MCF2L increases the Brdu positive cells, which further confirms that downregulation of MCF2L enhances HCC cell treated by sorafenib. Finally, in vivo animal experiments showed that the tumors formed by cells-MCF2L silenced are much smaller than that formed by control cells (Figure 2(e)) under the treatment of sorafenib.

Altogether, our results found that downregulation of MCF2L enhances HCC cell death induced by sorafenib.

### 3.3. MCF2L Is Involved in Ferroptosis of HCC Cell

**Figure 3:**
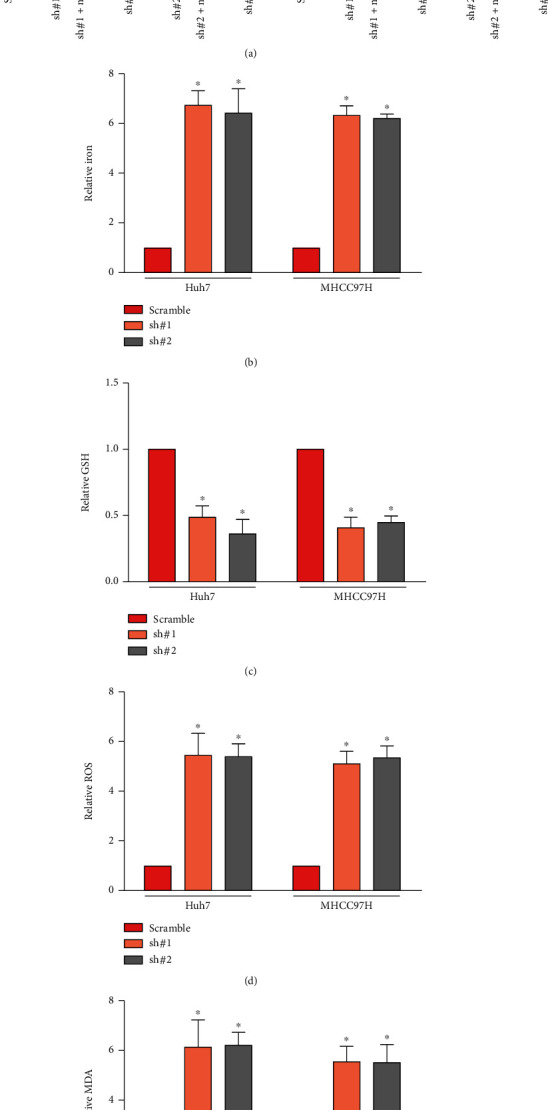
MCF2L is involved in ferroptosis of HCC cell. (a) The viability of MCF2L-konckdown cells can be rescued by ferroptosis inhibitor Ferrostatin-1, but not pan-caspase inhibitor Z-VAD-FMK, neccroptosis inhibitor necrosulfonamide, and autophagy inhibitor 3-MA using Huh7 (left panel) and MHCC97H (right panel) cell. (b) The relative Iron levels in indicated cells induced by sorafenib. (c) The relative levels of GSH in indicated cells induced by sorafenib. (d) The relative levels of ROS in indicated cells induced by sorafenib. (e) The relative levels of MDA in indicated cells induced by sorafenib. ^∗^*P* < 0.05.

Next, we clarify the forms of MCF2L downregulation involved in HCC cell death. Under treatment with sorafenib, the viability of MCF2L-konckdown cells can be rescued by ferroptosis inhibitor Ferrostatin-1, but not pan-caspase inhibitor Z-VAD-FMK, necroptosis inhibitor necrosulfonamide and autophagy inhibitor 3-MA (Figure 3(a)), suggesting that MCF2L might be involved in ferroptosis of HCC cells.

Ferroptosis is an oxidative, iron-dependent form of RCD, and accompanied by accumulation of ROS and lipid peroxidation products. So, we firstly detected the content of Iron. As shown in Figure 3(b), downregulation of MCF2L significantly reduces the content of Iron. During the ferroptosis, ROS can be scavenged by GPX4 by conversion of reduced GSH into the oxidized form GSSG [15, 16]. Therefore, we examined the level of GSH. The results showed that GSH content is significantly downregulated in MCF2L-silenced cells (Figure 3(c)). Then, we determined the content of ROS and MDA, the end products of lipid peroxidation. The results showed that downregulation of MCF2L substantially promotes the levels of ROS (Figure 3(d)) and MDA (Figure 3(e)), suggesting that downregulation of MCF2L promotes ferroptosis of HCC cells. Our analysis showed that MCF2L is involved in ferroptosis of HCC cells.

### 3.4. MCF2L Regulates PI3K/mTOR Pathway in a Rac1/RhoA-Dependent Manner

**Figure 4:**
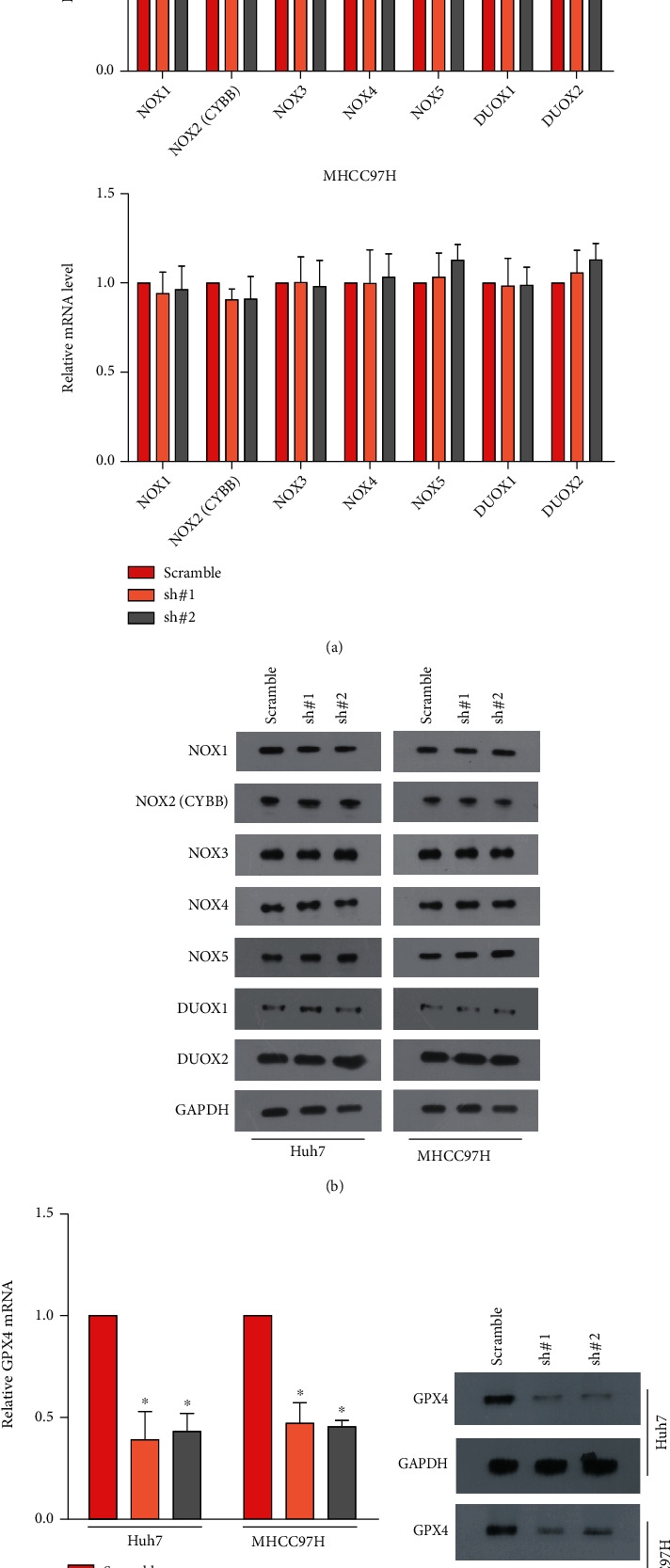
Downregulation of MCF2L significantly inhibits the levels of GXP4, but hardly changes the expression of NOXs (NOX1-5 and DUOX1-2) under the treatment of sorafenib. (a) The mRNA levels of NOXs. (b) The protein levels of NOXs. (c) The mRNA levels of GPX4. (d) The protein levels of GPX4.^∗^*P* < 0.05.

**Figure 5:**
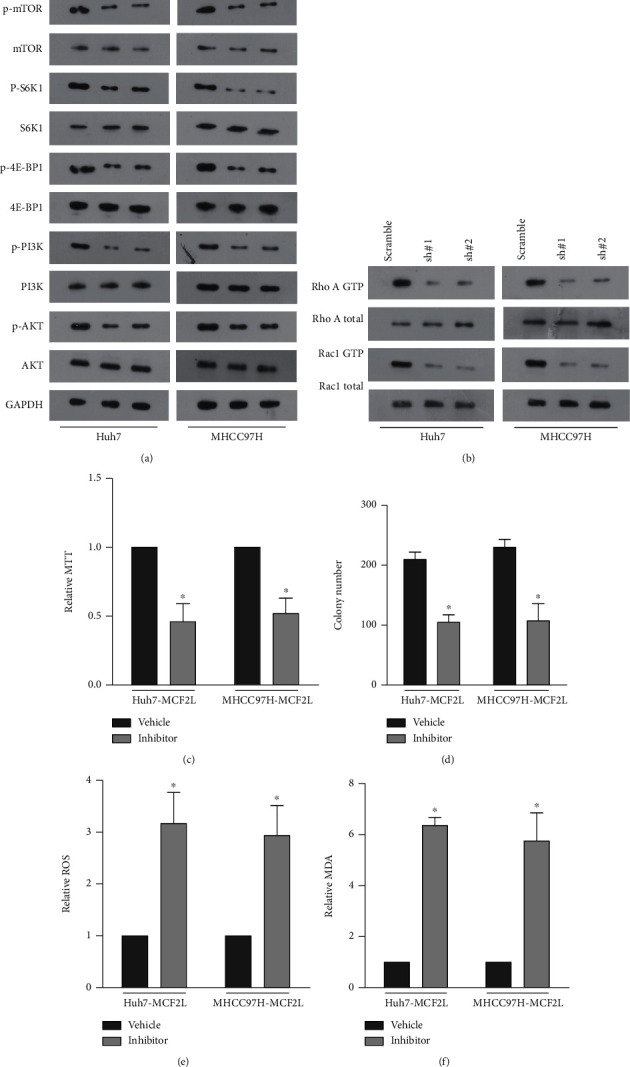
MCF2L regulates PI3K/mTOR pathway in a Rac1/RhoA-dependent manner. (a) Downregulation of MCF2L significantly inhibits the phosphorylation levels of PI3K (p-PI3K), AKT (p-AKT), mTOR (p-mTOR), and the levels of mTOR pathway mediators, p-S6K1 and p-4E-BP1. (b) Downregulation of MCF2L inhibits the activity of RhoA and Rac1. (c) MTT and (d) colony formation assay showed that inhibitors (NSC23766 and RhoGDIs) can significantly reversed the viability of HCC cells that promoted by overexpression of MCF2L compared with vehicle. (e) The relative ROS levels of HCC cells transfected with MCF2L plasmid treated using inhibitors (NSC23766 and RhoGDIs) or vehicle. (f) The relative MDA levels of HCC cells transfected with MCF2L treated using inhibitors (NSC23766 and RhoGDIs) or vehicle. ^∗^*P* < 0.05.

Since the amount of lipid ROS can be decided through impaired detoxification by GPX4 [10], or through production of superoxide and hydrogen peroxide by upregulation of NOXs [8]. We subsequently examined the levels of NOXs and GPX4. As shown in Figures 4(a) and 4(b), downregulation of MCF2L hardly changes the expression of all seven members of both mRNA and protein levels of the NOX protein family (NOX1-5 and DUOX1-2) in HCC cells. But, the mRNA and protein of GPX4 is significantly inhibited in MCF2L-konckdown cells (Figure 4(c) and 4(d)). It has been reported that GPX4 can be suppressed by the mTOR pharmacological inhibitor [17, 18]. And MCF2L works through RhoA/Rac1, and small GTPase family function upstream of PI3K by directly binding the PI3K catalytic subunit [19]. Therefore, we predicted that MCF2F regulated PI3K/mTOR pathway in a RhoA/Rac1-depent manner. As expected, downregulation of MCF2L significantly inhibits the phosphorylation levels of PI3K (p-PI3K), AKT (p-AKT), mTOR (p-mTOR), the levels of mTOR pathway mediators, p-S6K1 and p-4E-BP1, and the activity of RhoA and Rac1 (Figures 5(a) and 5(b)). Besides, the cells were treated with NSC23766 (a specific inhibitor of Rac) and RhoGDIs (a specific Rho inhibitor) in HCC cells transfected with MCF2L-overexpressed plasmids. The MTT and colony formation assay showed that inhibitors can significantly reverse the viability of HCC cells that promoted by overexpression of MCF2L compared with vehicle (Figures 5(c) and 5(d)). While, the inhibitors reverse the content of ROS and MDA that inhibited by overexpression of MCF2L compared with vehicle (Figures 5(e) and 5(f)).

Altogether, our analysis showed that MCF2L regulates PI3K/mTOR in a RhoA/Rac1 dependent manner to involve in ferroptosis of HCC cell.

## 4. Discussion

In the present study, we found that MCF2L is significantly upregulated in HCC tissues, and sorafenib can induce MCF2L upregulation. Downregulation of MCF2L promotes cell viability induced by sorafenib. Ferroptosis inhibitor can reverse the cell viability suppressed by sorafenib. Further analysis showed that downregulation of MCF2L can promote ferroptosis of HCC cells via PI3K/mTOR pathway in a Rac1/RhoA manner. Our finding demonstrated that targeting MCF2L may be a hopeful method to overcome sorafenib resistance in HCC.

Dixon et al. firstly discovered the phenomenon of ferroptosis, a new form of RCD different from autophagy, necrosis, and apoptosis [8]. Because of its nonapoptotic feature, ferroptosis-based cancer therapy is expected to overcome the disadvantages of traditional therapeutic strategy based on apoptosis pathways. Our study showed that MCF2L regulates the process of ferroptosis. When HCC cells were treated with sorafenib, downregulation of MCF2L induces cell ferroptosis and promoted cell death, which suggested that targeting MCF2L might be an effective method to overcome sorafenib resistance. Other molecules also have been studied to participate in ferroprosis in HCC. For example, Sun et al. found that metallothionein-1G facilitates sorafenib resistance through inhibition of ferroptosis, and genetic and pharmacological suppression of metallothionein-1G promoted the anticancer activity of sorafenib in vitro and in xenogenetic tumor models [20]. Liu et al. showed that circular RNA cIARS can promote the ferroptosis of HCC cells [21]. Wang et al. suggested that RNA binding protein DAZAP1 is demonstrated as a valid inhibitor of ferroptosis, and inhibition of DAZAP1 significantly reduces the proliferation and motility of HCC cells [22]. Li et al. showed that GALNT14 mediates ferroptosis of ovarian cancer via EGFR/mTOR signaling [23]. The study on ferroptosis provided more therapy strategies for drug resistance. And the molecule based on ferroptosis might be applicable in clinic in the short run.

Iron takes an important role in the execution of intracellular lipid peroxidation and ferroptosis. And the content of Iron at cellular and subcellular levels is an indispensable indicator to examine ferroptosis [24]. Our study showed that downregulation of MCF2L markedly increases Iron content. The accumulation of ROS and MDA are also the important indicators for indicators for ferroptosis, which can induce cell death through damaging biomolecules such as DNA/RNA and proteins [25]. Our analysis showed that downregulation of MCF2L can induce the accumulation of ROS and MDA.

The amount of lipid ROS can be decided through impaired detoxification by GPX4 [10], or through production of superoxide and hydrogen peroxide by upregulation of NOXs [8]. Yang et al. showed that low cell density is more likely to cause ferroptosis through upregulating NOX4 [26]. Besides, Poursaitidis et al. found that NOX4 suppression reduced cystine-deprivation induced cell death and lipid ROS, suggesting that NOX4 plays an important role in ferroptosis [27]. Nevertheless, our study showed that MCF2L hardly influence the levels of NOXs. But, MCF2L can regulate the levels of GPX4, which is a GSH-dependent enzyme and can reduce lipid hydroperoxides (L-OOH) to lipid alcohols (L-OH) [28]. Hence, we detected the levels of GSH and GPX4. The contents of GSH and GPX4 are both remarkedly downregulated in MCF2L-silenced cells.

Moreover, our findings demonstrated that MCF2L mediated ferroptosis via PI3K/mTOR pathway in a RhoA/Rac1 dependent manner. In recent decades, multiple study showed that mTOR pathway was discovered to regulate ferroptosis, for example, Zhang et al. discovered that benzopyran derivative 2-imino-6-methoxy-2H-chromene-3-carbothioamide (IMCA) can induce ferroptosis SLC7A11-depend through AMPK/mTOR pathway [29]; Liu et al. found that RSL3, a ferroptosis inducer, can suppress mTOR activity to induce the protein degradation of GPX4 in human pancreatic cancer cells [30]. In addition, RhoA/Rac1 is interacted with PI3K/AKT pathway. Zheng et al. showed that PI3K/Akt signaling cascade acts downstream of the RhoA/ROCK1 pathway [31].

However, the in vivo function of MCF2L on ferroptosis and the other mechanisms of ferroptosis in HCC should be clarified in our future research.

## Data Availability

The data that support the findings of this study are available from the corresponding author upon reasonable request.
